# Selected Pentacyclic Triterpenoids and Their Derivatives as Biologically Active Compounds

**DOI:** 10.3390/molecules30153106

**Published:** 2025-07-24

**Authors:** Zdeněk Wimmer

**Affiliations:** 1 Department of Chemistry of Natural Compounds, University of Chemistry and Technology in Prague, Technická 5, 16028 Prague, Czech Republic; zdenek.wimmer@vscht.cz or wimmer@biomed.cas.cz; 2 Isotope Laboratory, Institute of Experimental Botany of the Czech Academy of Sciences, Vídeňská 1083, 14220 Prague, Czech Republic

**Keywords:** pentacyclic plant triterpenoid, structural modifier, biological activity, cytotoxicity, antiviral activity, antimicrobial activity, signaling pathways, nano-material

## Abstract

Medicinal plants have been used in traditional medicines all over the world to treat human diseases throughout human history. Many of the medicinal plants have frequently become food and nutrition plants. A more sophisticated investigation resulted in discovering numbers of biologically important secondary metabolites of plants. Pentacyclic triterpenoids represent an important group of the plant secondary metabolites that have emerged as having top biological importance. While the most widespread plant triterpenoids and a majority of their semisynthetic derivatives have been reviewed quite often, other plant pentacyclic triterpenoids and their derivatives have so far been less frequently studied. Therefore, attention has been focused on selected pentacyclic triterpenoids, namely on arjunolic acid, asiatic acid, α- and β-boswellic acids, corosolic acid, maslinic acid, morolic acid, moronic acid, and the friedelane triterpenoids, and on different derivatives of the selected triterpenoids in this review article. A literature search was made in the Web of Science for the given keywords, covering the required area of secondary plant metabolites and their semisynthetic derivatives starting in 2023 and ending in February 2025. The most recently published findings on the biological activity of the selected triterpenoids, and on the structures and the biological activity of their relevant derivatives have been summarized therein. Even if cytotoxicity of the compounds has mainly been reviewed, other biological effects are mentioned if they appeared in the original articles in connection with the selected triterpenoids and their derivatives, listed above. A comparison of the effects of the parent plant products and their derivatives has also been made.

## 1. Introduction

Triterpenes and triterpenoids represent plant secondary metabolites, widely distributed in the plant kingdom, and numbering approximately 30 thousand different structures identified so far [1,2]. Among these plant products, pentacyclic triterpenes and triterpenoids have received priority attention due to the biological effects of different types [3]. Many of them have been considered for practical applications as therapeutic agents or dietary supplements all over the world [3]. These natural products can often be found in the medicinal plants used in traditional medicine. They have also become natural constituents of human diet, because they have been found in a great variety of fruits, vegetable oils, and cereals [4]. While their consumption in the Asian, African, and South American countries has been widely popular, in the Western world, their targeted application as food supplements started later, with the estimation of the individual average human consumption of triterpenes being calculated as approximately 250–400 mg per day, based on the country [5].

Beneficial health effects of fruits and vegetables have also been associated with the content of triterpenes and triterpenoids, in addition to other plant products [6]. The number of manuscripts and patents related to the biological activity and the therapeutic potential of triterpenes and triterpenoids and their semisynthetic derivatives is increasing, based on the results of searching the usual electronic databases. Pentacyclic triterpenoids and their derivatives display various types of biological effects that include anti-inflammatory [7], antioxidant [8], antiviral [9], anti-diabetic [10], anti-tumor [11], hepatoprotective [12], and cardioprotective [13] effects, as reviewed recently [1]. The evidence exists that pentacyclic triterpenes and triterpenoids have the potential to restore the vascular disorders associated with the hypertension, obesity, diabetes, and atherosclerosis [14]. They could be used in cancer therapy [15], as anti-ulcer drugs [16], and as the agents for the prevention and the treatment of metabolic diseases [1,3]. As a result of this general investigation, some triterpenoids have been included in clinical trials to evaluate their pharmacological potential, as mentioned in previous papers [1,17,18,19,20].

It has been broadly demonstrated in the literature that plant pentacyclic triterpenes, triterpenoids, and their various semisynthetic derivatives showed significant biological activity in the in vitro assays [21], as well as in some animal models [22]. However, their in vivo efficacy in humans has not yet been adequately studied, and it should be subjected to a much more detailed investigation to prove the applicability of these natural products and/or their semisynthetic derivatives in clinical practice. The whole process has been found to depend on many factors that include, namely, absorption, distribution, metabolism, and excretion (i.e., the ADME parameters) [23,24,25]. It has been demonstrated in the literature that the oral bioavailability barriers, the most important factor in the practical applicability of any biologically active agent, include solubility and/or dissolution, permeation, first-pass metabolism, and pre-systemic excretion from the intestine or from the liver [26].

The most serious threats all over the world are microbial and viral infections, and cancer, all of them representing diseases that are potentially fatal for humans [2,9,27,28]. Viral infections have been considered as the most important ones, and the most frequent diseases worldwide [9]. Generally, two categories of viruses invading the human body exist: (a) viruses present in the human body for a long time (e.g., herpes virus, hepatitis B/C virus, human influenza virus, etc.), and (b) viruses present in the animals that live close to humans (e.g., chickens, dogs, pigs, etc.). An additional important factor affecting human organisms has involved bacterial infections. These have become more and more dangerous for humans due to the existing widespread resistance of these pathogenic microorganisms to the currently used antimicrobial agents [28]. Finally, cancer is a serious and frequently occurring threat causing death to a significant proportion of the human population [2,27].

The number of people suffering from different cancers has been expected to rise together with the growing human population and its growing age. Changing lifestyles have contributed to the increasing cancer risk and represent the most important factor for humans all over the world. The leading factors causing death due to cancer have been varying across the countries and even within each country monitoring this development [3,4,5]. These factors have resulted in the finding that the early cancer stage detection methods should be applied more effectively, because the effective early stage treatment of cancers has not yet been achieved [6,7]. Cancer has often been understood as a highly heterogeneous disease consisting of different types of cells characterized by different molecular characteristics, and by the diverse therapeutic responses to the applied treatment agents [8,9,10]. To further discover new and efficient therapies for treating cancer as a global disease has presented a challenge for the researchers dealing with this type of investigation.

Natural products have always represented the most important source of products capable of becoming convenient candidates in the development of novel and nature-inspired drugs for different practical applications, including oncological ones [11,12,13,14]. Among a wide variety of natural products, pentacyclic triterpenoids have received emerging attention in the past two or three decades due to their multifunctional anticancer properties, in addition to a wide range of other important biological effects. Their low toxicity and their relatively widespread availability from sustainable natural sources have also contributed to the continuing success of plant pentacyclic triterpenoids in anticancer drug discovery, which has been widely documented in the literature so far [15,16,17,18,19,20,21,22].

Generally, plant pentacyclic triterpenoids are divided into several groups based on their general skeletons as lupanes, oleananes, ursanes, and friedelanes [26]. Each group consists of a number of pentacyclic triterpenoids, some of which frequently occur in nature, while others are either rare in nature or not frequently studied. The pentacyclic triterpenoids often studied and reviewed are betulinic acid [26,29,30], oleanolic acid [31], and ursolic acid [32,33]. A broad spectrum of the biological and medicinal properties of betulinic acid, particularly its potent cytotoxicity, has received significant attention in recent years. The cytotoxic characteristics of betulinic acid are controlled by the mitochondrial signaling pathways. Betulinic acid exhibits selectivity for the cancer tissues, leaving the normal cells and tissues untouched. This characteristic is particularly valuable in the chemo-resistant cases. Nevertheless, the medicinal potential of betulinic acid has been limited due to its poor solubility in water and a short half-life, leading to sub-optimal effectiveness. This issue is being tackled with a variety of nano-sized drug delivery systems, such as polymeric nanoparticles, magnetic nanoparticles, polymeric conjugates, nano-emulsions, liposomes, nano-suspensions, carbon nanotubes, and cyclodextrin complexes. The results reviewed so far summarized recent advances in nano-formulations developed for the delivery of betulinic acid to the target tissues and cells with an enhanced effectiveness [29]. As already mentioned above, the clinical application of betulinic acid is limited because of its low solubility in water, which impairs its distribution within the body. To meet this challenge, nano-emulsions have been developed to improve the bioavailability of such poorly soluble drugs [29,30]. Oleanolic acid and ursolic acid represent the pentacyclic triterpenoids, present in a variety of plants, namely in fruits and vegetables, and they were reviewed recently as well [31,32,33]. The natural plant foods that contain oleanolic acid and ursolic acid include apples, ginger, pears, tomatoes, grapes, strawberries, mangos, and olives (including olive oil, as the most important and widely distributed product), along with the culinary and therapeutically important herbs, namely rosemary, sage, oregano, ginseng, basil, fennel, garlic, and olive leaf. Applying in vitro preclinical investigations, researchers discovered a wide spectrum of biological properties for oleanolic acid and ursolic acid that include hepatoprotective, anti-inflammatory, anti-diabetes, antimicrobial, antihypertensive, gastroprotective, antihyperlipidemic, and anticancer effects [31,32,33].

Together with the increasing importance of supramolecular chemistry, attention has shifted from the application of traditional polymeric materials to the design and development of multifunctional supramolecular materials [34]. Natural small molecules, mostly of plant origin, displaying self-assembly characteristics have been preferred over synthetic molecules. Natural products have showed lower toxicity, and better biocompatibility, biodegradability, and bioactivity in comparison with synthetic compounds. The pentacyclic triterpenoids have become promising natural products for the construction of supramolecular materials, because they possess numerous modification sites, and display generally useful functions. At present, many supramolecular materials based on different triterpenoids have been successfully developed and applied in practice [34]. They have been shown to play important roles in tissue engineering, biomedicine, food science, and in flexible electronics. Current findings of the investigation of the triterpenoid-based supramolecular materials that include low-molecular-weight gels, nano-carriers for drug delivery, chiral self-assembled materials, and emulsion gels, and focus mainly on the preparation methods of such materials and on the nano-assembly processes, structural characteristics, and applications, were described recently [34]. Nano-assembly also appears during the investigation of the selected pentacyclic triterpenoids reviewed here. A relationship between the biological activity of the reviewed compounds and their ability to form various nano-assemblies will also be mentioned several times in this review.

This review article will not focus on the frequently occurring triterpenoids, betulinic acid, oleanolic acid, and ursolic acid and their derivatives, for which many original and review papers exist to document their importance in medicinal chemistry, pharmacology, and medicine [35,36,37,38]. Here, attention has been paid to the less frequently used pentacyclic triterpenoids, often displaying biological effects surpassing those of the frequently studied triterpenoids. The main objective of this review article has been focused on several less frequently studied plant pentacyclic triterpenoids, their derivatives, and their importance in the medicinal and supramolecular research. The pentacyclic triterpenoids reviewed here are arjunolic acid, asiatic acid, α- and β-boswellic acids, corrosolic acid, maslinic acid, morolic acid and moronic acid, and the friedelane triterpenoids.

## 2. Arjunolic Acid

The most recent review papers dealing with arjunolic acid were published several years ago [39,40]. Arjunolic acid was found in the *Terminalia arjuna* tree, from which it was extracted in the form of a saponin [40]. It has displayed anticancer, antioxidant, anti-inflammatory, hypoglycemic, anti-diabetic, cytoprotective, antibacterial, antifungal, and cardioprotective activity, as well as other biological effects [39,40,41]. Recently, the semisynthetic derivatives of the natural arjunolic acid were prepared and studied. Such derivatives often display biological effects and profiles that are more useful for the potential biological and/or pharmacological applications than those of the parent arjunolic acid.

**Figure 1:**
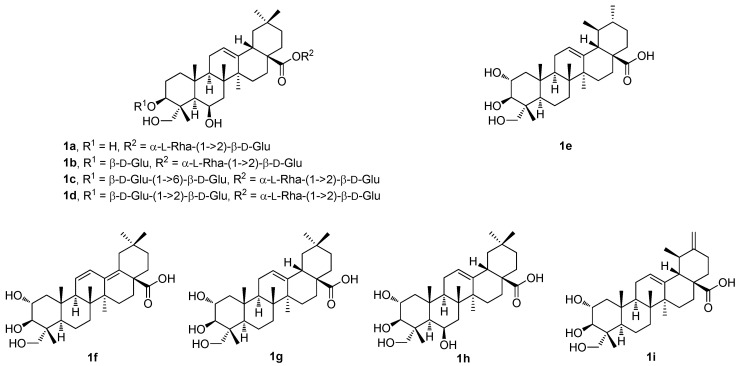
The structures of the pentacyclic triterpenoids and their glycoside derivatives isolated from *Cryptolepis buchananii* fruits.

Thus, four undescribed pentacyclic triterpenoid glycosides (**1a**–**1d**; Figure 1) and five known pentacyclic triterpenoids (**1e**–**1i**; Figure 1) were isolated from the methanol extract of the *Cryptolepis buchananii* fruits [42]. The structures of the compounds were elucidated. They were identified as uncargenin-C(28)-*O*-α-l-rhamnopyranosyl-(1→2)-β-d-glucopyranosyl ester (**1a**), 3-*O*-β-d-glucopyranosyluncargenin C(28)-*O*-α-l-rhamnopyranosyl-(1→2)-β-d-glucopyranosyl ester (**1b**), 3-*O*-β-d-glucopyranosyl-(1→6)-β-d-glucopyranosyl-6β,23-dihydroxyursolic acid 28-*O*-α-l-rhamnopyranosyl-(1→2)-β-d-glucopyranosyl ester (**1c**), 3-*O*-β-d-glucopyranosyl-(1→2)-β-d-glucopyranosylasiatic acid 28-*O*-α-l-rhamnopyranosyl-(1→2)-β-d-glucopyranosyl ester (**1d**), asiatic acid (**1e**), 2α,3β,23-trihydroxyoleana-11,13(18)-dien-28-oic acid (**1f**), arjunolic acid (**1g**), 6β-hydroxyarjunolic acid (**1h**), and actinidic acid (**1i**), based on the analysis of their HRMS (ESI), and of the 1D and 2D NMR spectra [42]. The compounds **1a**–**1i** (Figure 1) were screened for their inhibition of nitric oxide (NO) production in the LPS-activated RAW264.7 cells [42]. The inhibition of NO production caused by the compounds **1a**–**1i** was evaluated at sequentially diluted concentrations. Compound **1e** showed the strongest inhibitory activity (IC_50_ = 8.91 μM) among the studied series of compounds. Its inhibitory activity was even higher than that of the positive control compound, dexamethasone (IC_50_ = 14.05 μM) (Table 1). The other compounds of this series showed significant effects within the range of IC_50_ = 18.78–37.57 μM. These results suggested that the pentacyclic triterpenoids bearing either the 3β,23-dihydroxyoleanan-12-en-28-oic acid skeleton or the 2α,3β,23-trihydroxyursan-12-en-28-oic acid skeleton, and their glycosyl derivatives formed at the C(3) and the C(28) carbon centers, may play important roles in the inhibition of NO production [42].

**Figure 2:**
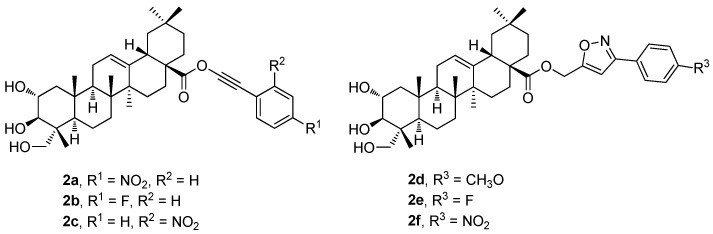
The phenyl acetylene and the phenyl isoxasole derivatives (**2a**–**2f**) of arjunolic acid (**1g**).

The new phenyl acetylene and the isoxazole analogs of arjunolic acid were designed, synthesized, and evaluated (**2a**–**2f**; Figure 2) as potential inhibitors of tyrosinase and α-glucosidase [43]. The tested compounds exhibited stronger inhibitory effects than the reference drug or the parent arjunolic acid (**1g**) (Table 2). Compound **2e** displayed the most potent tyrosinase inhibitory effect (IC_50_ = 14.3 ± 7.6 μM) exceeding about three times the effect of the reference drug, kojic acid (IC_50_ = 41.5 ± 1.0 μM). Compound **2f** (IC_50_ = 14.5 ± 0.15 μM) displayed a potent α-glucosidase inhibitory effect with the IC_50_ value comparable to that of the reference compound (acarbose; IC_50_ = 10.4 ± 0.06 μM). Therefore, compounds **2e** and **2f** became promising candidates for more detailed studies to be conducted in the future [43].

Table 2 shows the results of the α-glucosidase and tyrosinase inhibitory effects of the compounds **1g** (Figure 1) and **2a**–**2f** (Figure 2) in comparison with the effect of the reference compound (acarbose). In the evaluation of the α-glucosidase inhibitory effect, arjunolic acid (**1g**; IC_50_ > 600 μM; Table 2) displayed a very low α-glucosidase inhibitory activity. The results also show that the isoxazole analogs (**2d**–**2f**) displayed a strong inhibitory activity against the α-glucosidase enzyme compared to the effect of the phenyl acetylene counterparts (Table 2). Among the isoxazole derivatives, compound **2f** was the most active one (IC_50_ = 14.5 ± 0.15 μM). Nevertheless, the isoxazole-bearing molecules displayed a significant inhibition of the enzyme comparable to that of acarbose. Compound **2d** displayed inhibitory activity, which was the highest one among the series of the phenyl acetylene analogs. Compounds **2a**–**2f** showed higher inhibitory effects than **1g**. The results of the tyrosinase inhibitory effects of **1g** (Figure 1) and **2a**–**2f** (Figure 2) in comparison with the effect of the reference compound (kojic acid) are also summarized in Table 2. The results revealed that **2a**–**2f** showed higher tyrosinase inhibitory effects than the parent arjunolic acid (**1g**). The isoxazole-bearing compounds (**2d**–**2f**) displayed effects more than three times higher than **1g**. The compounds **2d**–**2f** showed higher tyrosinase inhibitory effects than the positive control, kojic acid (IC_50_ = 41.5 ± 1.0 μM). This finding revealed that the potency of the tested compounds is dependent on their functionalities. The decreasing order of the tyrosinase inhibitory activity of the compounds was found and evaluated in the order of **2e** > **2f** > **2d** > **2b** > **2c** > **2a** > **1g**.

**Figure 3:**
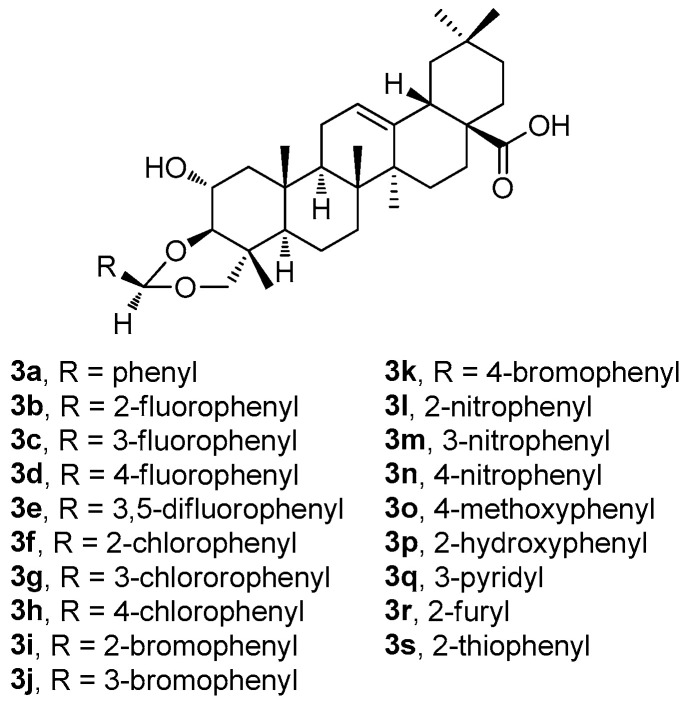
The structures of the arjunolic acid acetals (**3a**–**3s**).

Recently, Indian researchers synthesized and characterized a series of novel arjunolic acid acetals (**3a**–**3s**; Figure 3) [44]. Cytotoxicity of the synthesized acetals was investigated against sixty cell lines from nine different types of cancers at the National Cancer Institute [44]. Compounds **3b**, **3d**, **3i,** and **3r** demonstrated significant cytotoxicity in colon cancer, melanoma, renal cancer, and breast cancer [44]. The studies of the two most active compounds (**3i** and **3r**) revealed cell cycle arrest in the G2/M phase, which induced ROS generation in the cells, finally leading to cell death. In addition, compound **3i** showed better selectivity for the tumor cells in comparison with the non-malignant cells [44].

Among compounds **3a**–**3s** (Figure 3), the selected compounds **3b**, **3d**, **3i,** and **3r** have proven their ability to become promising agents due to their potent anticancer activity in almost all tested cell lines, most remarkably in colon cancer, melanoma, and leukemia cell lines (Table S1, Supplementary Material). The selected results of testing in several cancer cell lines resulted in the conclusion that **3r** was the most active compound of this series (Table 3). However, based on the additional tests performed, and on the subsequent dose-dependent evaluation in the colon cancer cell line CT-26, the results revealed that **3i** was the most potent compound of this series (**3i**; IC_50_ = 2.53 μM). Compound **3i** also showed a better profile in treating the non-malignant cells than **3r** [44]. A propidium iodide (PI) uptake assay revealed a higher proportion of the PI-positive cells in the groups treated by **3i** compared to the untreated controls, indicating the increasing cytotoxicity (cf. graphical figures in [44]). A flow cytometry-based cell cycle analysis further resulted in a finding that **3i** was capable of inducing the G2/M phase cell cycle arrest. Additionally, the treatment of the CT-26 cancer cells with **3i** resulted in elevated ROS levels, suggesting that the mechanism of action involves ROS-mediated pathways. Based on these findings, compound **3i** represented the most promising compound displaying cytotoxicity in the CT-26 colon cancer cell lines among the whole series of the prepared arjunolic acid acetals [44]. It can be proposed that **3i** should be further studied for structural optimization for the development of practically applicable therapeutic agents to treat colon cancer [44].

**Figure 4:**
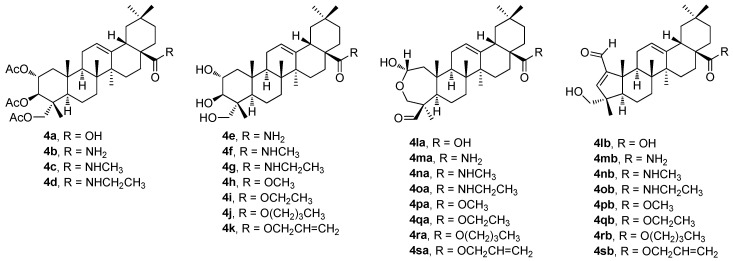
The structures of the prepared derivatives (**4a**–**4sb**) of arjunolic acid (**1g**).

Another novel series of derivatives of **1g**, containing a pentameric A-ring with an enal moiety combined with the additional modifications at the C(28) carboxyl group, was designed (**4a**–**4sb**; Figure 4) [45]. The biological investigation focused on the viability of the human cancer cell lines and on that of the non-malignant cells. It was evaluated in order to identify the most promising compounds of this series (Table 4a). A structure–activity relationship analysis was performed, resulting in identifying the most active derivative (**4qb**). It also showed reasonable selectivity between the malignant cells and the non-malignant fibroblasts (Table 4b) [45]. The molecular mechanism of action of cytotoxicity in the PANC-1 (human pancreatic cancer) cells was further studied with **4qb**, and the results showed that **4qb** induced cell cycle arrest at the G0/G1 phase. It significantly inhibited the wound closure rate of the PANC-1 cancer cells in a concentration-dependent manner. In addition, **4qb** synergistically increased the cytotoxicity of gemcitabine, especially at a concentration *c* = 0.24 μM. Moreover, based on a preliminary pharmacological study, **4qb** was found to display no cytotoxicity in vivo at lower doses. Taken together, these findings suggested that **4qb** might represent a valuable compound for treating pancreatic cancer [45]. Subsequent studies were recommended to explore the full potential of the compound **4qb**.

To summarize the structure–activity relationships of the reviewed derivatives of arjunolic acid (**1g**), several compounds should be pointed out. Arjunolic acid (**1g**) caused an inhibition of the nitric oxide (NO) production in the LPS-activated RAW264.7 cells in comparison to the positive reference compound (dexamethasone). Compound **2f** was the most active of the series of the phenyl acetylene and phenyl isoxasole derivatives (**2a**–**2f**) of **1g** in the study of the α-glucosidase and tyrosinase inhibitory activity. Compound **2f** showed α-glucosidase inhibitory activity when compared with acarbose, the positive reference compound, and it displayed better tyrosinase inhibitory activity than kojic acid. However, several compounds of this series showed better tyrosinase inhibitory activity than **1g**, without having α-glucosidase inhibitory activity. Four compounds (**3b**, **3d**, **3i,** and **3r**) from the series of the arjunolic acid acetals showed the most promising activity values in a panel of nine different cancers consisting of 60 different cancer cell lines. Compounds **3i** and **3r** were evaluated as the compounds of priority importance among the selected sub-series of four compounds subjected to the given detailed investigation. Finally, the derivatives of **1g** containing a pentameric A-ring with the enal moiety (**4a**–**4sb**) were initially tested in the PANC-1 and HT-29 cancer cell lines. Based on the results obtained, cytotoxicity of the selected compounds **4pb**–**4sb** was investigated in several cancer cell lines (melanoma and lung), and in the non-malignant human fibroblasts, showing the potential of all selected compounds to treat these cancers. However, the disadvantage of the compounds **4pb**–**4sb** consisted in their relatively high toxicity in the non-malignant human fibroblasts as well.

## 3. Maslinic Acid, Asiatic Acid, and Corosolic Acid

Hawthorn, a medicinal food homology plant, belongs to the *Crataegus* genus in the Rosaceae family, and it represents a highly valuable plant for various applications [46]. Due to its long history of medicinal use, remarkable effects, and safety, hawthorn has been paid considerable attention. It has been found to play an important role in cancer treatment as well. The cytotoxic ingredients in hawthorn have been predicted, identified, and analyzed by modern pharmacology technology, which has obtained this knowledge from traditional Chinese medicine [46]. The performed studies showed that the ingredients found in hawthorn, namely vitexin, isoorientin, ursolic acid, and maslinic acid, and the hawthorn extracts themselves as well, are capable of modulating the cancer-related signaling pathways effectively, and are capable of demonstrating cytotoxic properties via diverse mechanisms. Maslinic acid was also found in olive oil [47]. The isolated maslinic acid displayed antioxidant activity and an antimicrobial effect, namely in *Streptococcus pyogenes*, a G^+^ microorganism [47]. The extract of *Crossopteryx febrifuga* (Rubiaceae), a plant widely used in traditional African medicine, was used to treat trypanosomiasis [48]. However, the analysis of the extract resulted in the identification of approximately ten main components, of which maslinic acid and corosolic acid are the parts contributing to the anti-trypanosomal activity [48].

**Figure 5:**
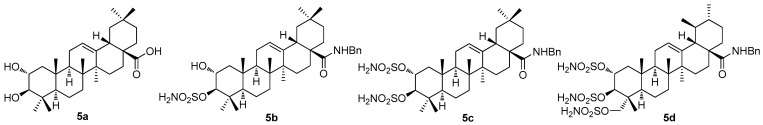
The structures of maslinic acid (**5a**), its sulfamated derivatives **5b** and **5c**, and the sulfamated derivative **5d** of asiatic acid (**1e**).

Recently, the cytotoxic, anti-diabetic, antibacterial, and anti-parasitic effects of maslinic acid (**5a**; Figure 5) have been proven by investigation [49]. Concerning cancer research, maslinic acid (**5a**) was found to cause apoptosis by activating the p53/JNK, Bcl-2, caspase-3, caspase-8, and caspase-9 pathways [50]. Maslinic acid (**5a**) and its analogs were tested against the 3D-QSAR model using the human breast cancer cell line (MCF7) to determine the in vitro cytotoxicity of the studied compounds [51]. Maslinic acid and its derivatives were also found to form supramolecular nano-assemblies [49]. Generally, nano-assemblies are ubiquitous in biological systems. Due to the variations in the triterpenoid backbone structures and different orientations of the functional groups in space, the self-assembly characteristics vary in the different triterpenoid-based molecules. The self-assembled nanostructures have been exploited for the entrapment of the biologically and/or pharmacologically active agents, including drugs displaying cytotoxicity, and they can also be used as carriers of the drug molecules. The nano-assemblies are useful for the generation of thermochromic and hybrid materials, recyclable heterogeneous catalysts, agents responsible for removing toxic chemicals, agents responsible for the formation of liquid crystals, etc. [52]. Maslinic acid (**5a**) was found to self-assemble spontaneously in aqueous-organic media, yielding nano-assemblies mostly insoluble in water due to the absence of an adequate number of polar functional groups and due to the presence of a long lipophilic backbone [53]. The potential cytotoxicity and antimicrobial activity of the nano-assemblies of maslinic acid and those of its derivatives were intensively investigated, with promising results [49].

Maslinic acid (**5a**) has a variety of biological activities, such as anti-tumor, hypoglycemic, anti-inflammatory, and anti-parasitic effects. In order to enhance the biological activity of maslinic acid, numerous structural modifications have been designed, and the more valuable maslinic acid derivatives were synthesized [54]. It has been found that the derivatives of maslinic acid always showed better characteristics than those of oleanolic acid, and the derivatives of corosolic acid similarly showed better characteristics than those of ursolic acid [55].

Most of the authors of the papers dealing with pentacyclic triterpenoids performed their investigation on more than a single triterpenoid, which has made this review paper rather difficult to complete. A recent investigation by German researchers has dealt with the sulfamated derivatives of maslinic acid (**5a**) and asiatic acid (**1e**) [56]. The study involved not only the triterpenoids **5a** (Figure 5) and **1e** (Figure 1), but also involved other triterpenoids (oleanolic acid, betulinic acid, and platanic acid), focusing on the inhibitory capability of the selected pentacyclic triterpenoids on diverse isoforms of the human carbonic anhydrases (*h*CAs) [56]. These enzymes are responsible for the catalysis of the reversible hydration of carbon dioxide in the blood, yielding bicarbonates and protons. They are important in physiological processes that include respiration, gluconeogenesis, adipogenesis, and numerous other biosynthetic reactions [57]. The human carbonic anhydrases have been subjected to many types of investigations as the therapeutic targets for a wide variety of diseases, e.g., edema, glaucoma, epilepsy, obesity, inflammatory diseases, neuropathic pain, Alzheimer’s disease, oxidative stress, and hypoxic tumors [57,58]. The existing relationships between the cancers and the individual carbonic acid anhydrase isoforms have already been described [59].

The synthesis of compounds **5b**–**5d** (Figure 5) involved several synthetic steps starting from the parent triterpenoid acids **5a** (Figure 5) and **1e** (Figure 1). The whole synthetic process was described in the original literature [56]. The inhibition assays using **5b**–**5d** (Figure 5) against *h*CAs I, II, VA, and IX resulted in remarkable outcomes (Table 5). In summary, **5d** was found to represent a robust and selective *h*CA VA inhibitor, which was the most important finding for further exploration of its therapeutic applications [56].

**Figure 6:**
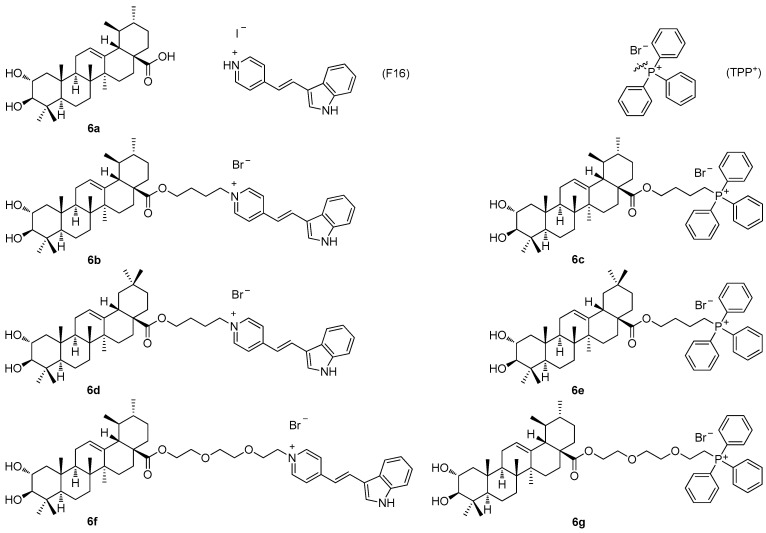
The structures of corosolic acid (**6a**), the compound F16, the cation (TPP^+^), and the conjugates **6b**–**6g** derived from **6a** and **5a**.

Another recent paper of this series described the investigation of the cytotoxic profile, and anti-proliferative and mitochondrial effects of the conjugates of maslinic acid (**5a**; Figure 5) and corosolic acid (**6a**; Figure 6) with the mitochondriotropic and lipophilic triphenylphosphonium (TPP^+^) cationic species (TPP^+^Br^−^) and with the (*E*)-4(1*H*-indol-3-ylvinyl)-*N*-methylpyridinium iodide (known as F16) [60]. Maslinic acid (**5a**; Figure 5) and corosolic acid (**6a**; Figure 6), chosen as the investigation objects, were synthesized from the commercially available oleanolic acid and ursolic acid [60]. The investigation of the cytotoxicity of the triterpenoid-based derivatives with the F16 and the TPP^+^ species in the six tumor cell lines demonstrated a comparable synergistic effect in cytotoxicity, which was most pronounced in the case of the mammary adenocarcinoma cells (MCF-7), and the leukemia cells (Jurkat and THP-1) (Table 6) [60]. The corosolic acid and the maslinic acid conjugates **6b**–**6g** (Figure 6) showed changes in the tumor cell cycle phases when present in much lower doses than their natural triterpenoid precursors **6a** and **5a**. A treatment of the tumor cell lines with these compounds resulted in a cell cycle arrest in the G1 phase, and in an increase in the cell population in the subG1 phase. The cationic derivatives of the acids were found to show higher activity than their precursors as inducers of the hyperproduction of the reactive oxygen species (ROS). The observed cytotoxic effects of the F16 and the TPP^+^ triterpenoid conjugates **6b**–**6g** resulted in a finding, on the basis of which the conjugates **6b**–**6g** were evaluated as agents capable of initiating mitochondrial dysfunction, because they caused an effective decrease in the mitochondrial potential in the isolated rat liver mitochondria [60]. Cytotoxicity, the anti-proliferative action, and the mitochondrial effects of **6b**–**6g** were found to be dependent on the type of the cationic group [60]. The disadvantage of this series of compounds consisted in their cytotoxicity in the non-malignant cells, which strongly limited the importance of this series of compounds (Table 6).

**Figure 7:**
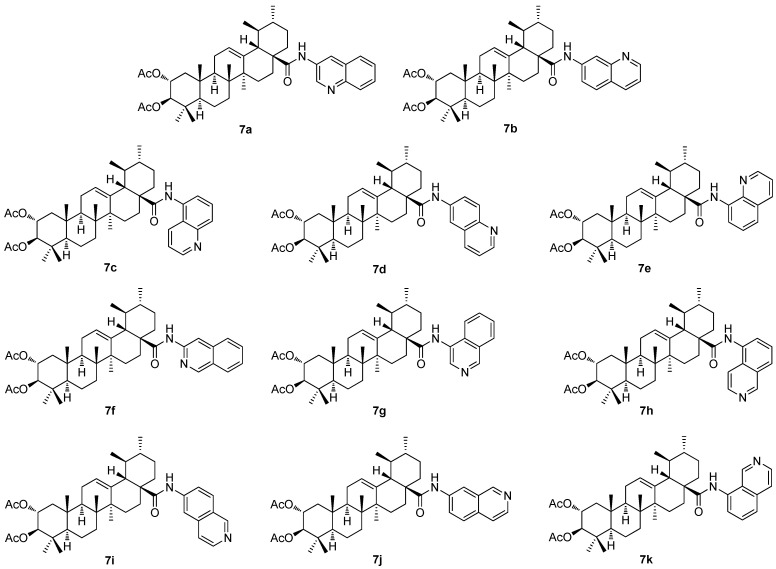
The structures of the quinoline derivatives (**7a**–**7e**) and the isoquinoline derivatives (**7f**–**7k**) of corosolic acid (**6a**).

The investigation of the (iso)quinolinyl amides of the acetylated triterpenoids, performed by the German authors, resulted in a finding that the studied compounds displayed excellent cytotoxicity and, under specific conditions, high selectivity [61,62,63]. In particular, the derivatives of maslinic acid (**5a**; Figure 5) or asiatic acid (**1e**; Figure 1) showed promising results [56,64,65]. Subsequently, the effect of different arrangements of the two methyl groups in ring E, compared to maslinic acid (**5a**), and the absolute configuration of the hydroxyl groups in ring A, compared to asiatic acid (**1e**) was investigated. Corosolic acid (**6a**; Figure 6) was chosen as the starting material [63]. Corosolic acid (**6a**) was acetylated and transformed into the corresponding quinolinyl amides **7a**–**7e** (Figure 7) and isoquinolinyl amides **7f**–**7k** (Figure 7) using various amino–(iso)quinolines for the systematic investigation. Their analysis using the SRB assays revealed that several of the synthesized amides exhibited significant cytotoxicity in a range of five human cancer cell lines, using the non-malignant human fibroblasts (CCD18Co) as the reference cells (Table 7). Notably, compound **7b**, the 7-aminoquinoline derivative, emerged as the most potent compound, demonstrating not only high cytotoxicity but also good selectivity in the tumor cells and a remarkable ability to overcome drug resistance. The highest selectivity index was obtained for **7b** (the 5-aminoquinoline derivative) and the HT29 colorectal carcinoma cells with an SI > 74.5, and for **7k** (the 8-isoquinoline derivative) with an SI > 58.5, while the reference compound (doxorubicin) showed an SI = 2.9 under the same conditions [63].

The structure–activity relationships of the reviewed derivatives of maslinic acid (**5a**), asiatic acid (**1e**), and corosolic acid (**6a**) were evaluated together, because these pentacyclic triterpenoids were often studied together in the original literature. The sulfamated derivatives of maslinic and asiatic acid were almost inactive in the inhibition of the human carbonic anhydrase isoforms. The only compound, **5d,** derived from asiatic acid inhibited the human carbonic anhydrase isoform *h*CA VA with comparative activity to the positive reference compound (acetazolamide). A series of the ionic derivatives of maslinic and corosolic acid resulted in a finding that several compounds of this series (**6a**–**6g**) showed high cytotoxicity in the MCF7, Jurkat, and THP-1 cancer cell lines. The compounds of this series also showed relatively high toxicity in the non-malignant cells, and this finding eliminated these compounds from subsequent investigation. Cytotoxicity of the quinoline and the isoquinoline derivatives (**7a**–**7k**) of corosolic acid (**6a**) was tested in several cancer cell lines. The investigation revealed that **7b** was the most active compound in treating the HT-29 cancer cell line, showing a cytotoxicity comparable to that of doxorubicin.

## 4. Moronic Acid and Morolic Acid

**Figure 8:**
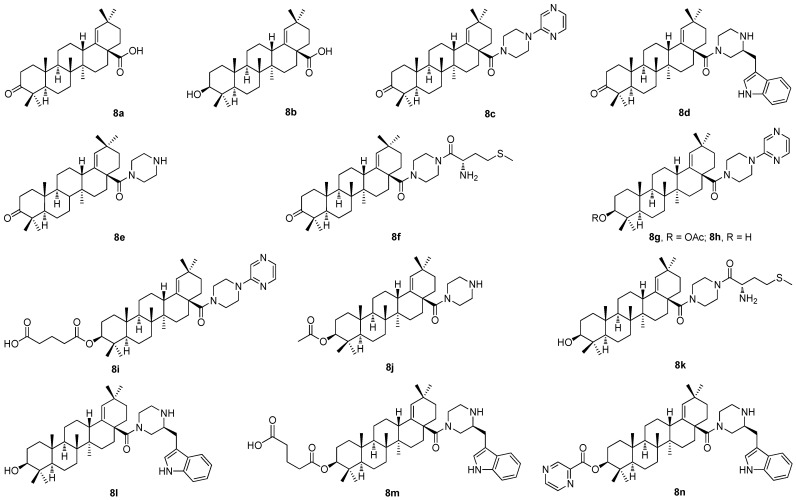
The structures of the amides of moronic acid and of morolic acid (**8a**–**8n**).

Moronic acid (**8a**) and morolic acid (**8b**) are rare plant secondary metabolites belonging to the group of pentacyclic triterpenoids with oleanane skeletons (Figure 8). Both triterpenoids display many biological effects [66,67,68,69,70,71,72,73]. Moronic acid (**8a**) has become an alternative agent for the treatment of the type 2 diabetes, which is its most important effect [74]. In turn, morolic acid (**8b**) displays cytotoxicity, anti-HIV and anti-HSV activity, and anti-inflammatory and anti-diabetic effects [75]. The significant therapeutic properties of morolic acid (**8b**) are desirable in the context of biological and pharmacological research, and drug development, but the low accessibility of **8b** from natural resources has limited its applications so far. The articles dealing with the investigation of morolic acid (**8b**) are also rare. Nevertheless, morolic acid (**8b**) seems to be important in treating leishmaniasis, a tropical infectious disease [76,77]. Morolic acid (**8b**) and its simple derivative, 3-*O*-caffeate, were successfully applied during the investigation of metabolic diseases including diabetes and obesity, and cardiovascular and kidney diseases [78]. However, the amide derivatives of morolic acid investigated within the research of my team seem to be biologically important compounds that do show biological activity [79,80].

Morolic acid (**8b**) and moronic acid (**8a**), like all similar plant secondary metabolites, appear in nature in the form of conjugates, mostly with oligosaccharides, i.e., saponins. In this form, their solubility in water or in physiological media is acceptably high [81]. Natural triterpenoid saponins have recently been investigated as potential SARS-CoV-2 inhibitors [82]. In turn, saponins can be split relatively easily by a chemical method, by liberating the non-polar aglycone (sapogenin), mainly during the isolation processes. An efficient way to make the investigation of the biological activity of these triterpenoids easier, is to prepare their semisynthetic conjugates, both of a polar or of a non-polar nature, for different types of biological applications.

Moronic acid (**8a**) and morolic acid (**8b**) were subjected to structural modifications within the investigation performed by my research team [79]. Several structural modifiers, namely, piperazine-, pyrazine-, 1*H*-indole-, and l-methionine-based compounds, were used, and a series of compounds (**8c**–**8n**; Figure 8) was prepared [79]. The derivation was targeted to designing and preparing novel compounds capable of nano-assembly and/or displaying cytotoxicity. The formation of different types of nanostructures has been proven for several novel target compounds that formed different types of nanostructures, either in chloroform or in water [79]. Cytotoxicity of **8c**–**8n** was investigated using three cancer cell lines and the normal human fibroblasts as the reference non-malignant cells, and the results are summarized in Table 8 [79].

**Figure 9:**
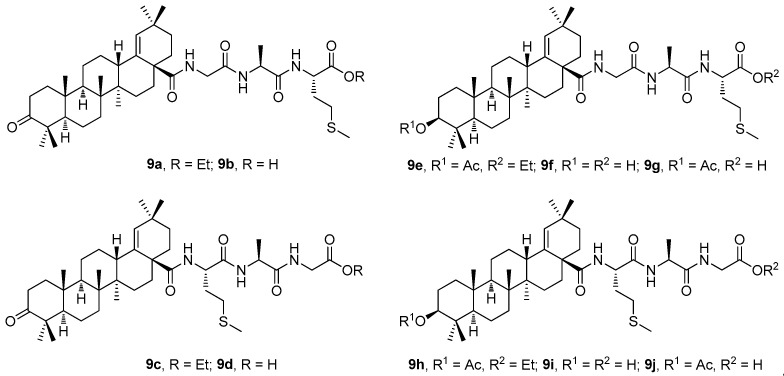
The structures of the compounds **9a**–**9j**.

Another series of the amides of moronic acid (**8a**) and of morolic acid (**8b**) was designed and synthesized using the tripeptides MAG and GAM [80]. Two required tripeptides were synthesized by a stepwise chain elongation of the ethyl esters of either glycine or l-methionine at their *N*-termini using the Boc-protected amino acids in each step [80]. The tripeptides GAM and MAG were then used in the synthesis of **9a**–**9j** (Figure 9), the derivatives of **8a** and **8b** [80]. The target compounds and their synthetic intermediates were subjected to the investigation of their antimicrobial, antiviral, and cytotoxic activity.

Table 9 summarizes the selected results of the antimicrobial, antiviral, and cytotoxicity effects of the most promising compounds of the studied series [80]. The presented results demonstrate a remarkable selectivity in the biological effects of the compounds **9a**–**9j** (Figure 9). Compound **9d** displayed a higher antimicrobial activity on *S. aureus* than the parent compound **8a** (Table 9). In the anti-HIV-1 tests, compounds **9g**, **9i,** and **9j** were less potent than the parent compound **8b**. However, in the anti-HSV-1 tests, compounds **9g**, **9i,** and **9j** showed higher selectivity of their antiviral effect, because they displayed no cytotoxicity in a comparison with the parent **8b** (Table 9). Finally, in the cytotoxicity assays, the effect of **9h** was superior to that of its parent structure **8b** (Table 9) [80].

The structure–activity relationships of the derivatives of moronic acid (**8a**) and of morolic acid (**8b**) revealed that the compounds **8k** and **9h** showed an acceptable cytotoxicity in several cancer cell lines (CCRF-CEM, HeLa, MCF7, and G-361). The compound **9h** showed a slightly better profile than **8k,** showing selectivity index values in the listed cancer cell lines as SI > 4.0, >6.0, >6.5, and >6.3, respectively, while being non-toxic in the normal human fibroblasts (IC_50_ > 50 µM). Moreover, the antimicrobial and the antiviral activity of the compounds **9a**–**9j** was studied. Compound **9d** inhibited *S. aureus* at 100%. Compounds **9g**, **9i,** and **9j** showed anti-HIV-1 and/or the anti-HSV-1 activity with selectivity index values comparable to those of **8a** and **8b** or even better than the selectivity index values of **8a** and **8b**.

## 5. Boswellic Acids

**Figure 10:**
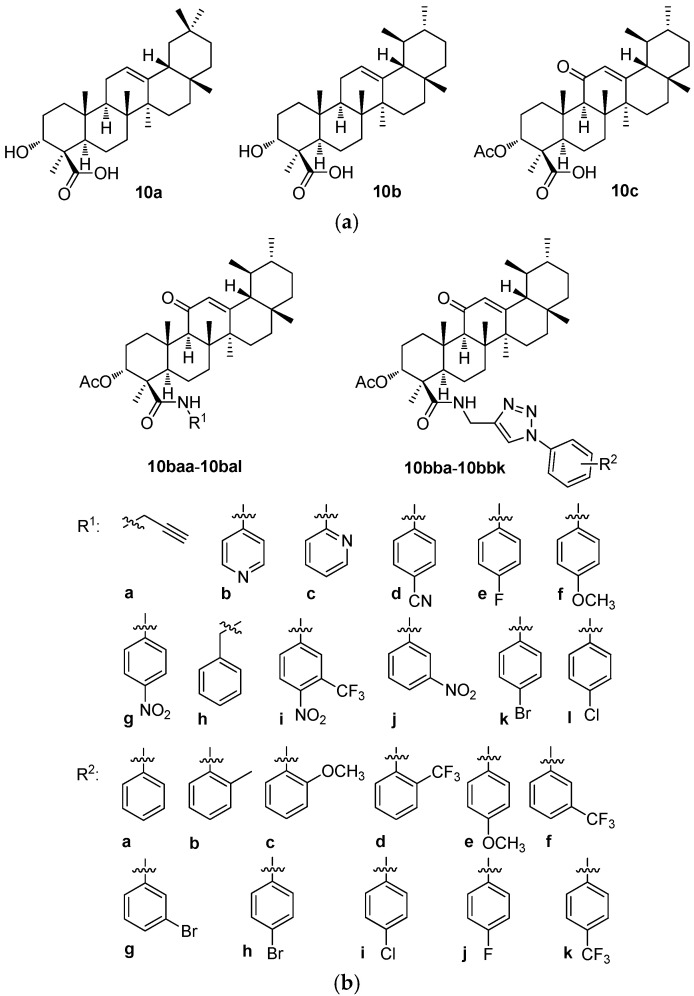
(**a**). The structures of α-boswellic acid (**10a**), β-boswellic acid (**10b**), and 3-*O*-acetyl-11-keto-β-boswellic acid (β-AKBA; **10c**). (**b**). The structures of the compounds **10baa**–**10bal** and **10bba**–**10bbk** derived from **10c**.

The boswellic acids form an isomeric pair of pentacyclic triterpenoids, α-boswellic acid (**10a**; the oleanane-type triterpenoid) and β-boswellic acid (**10b**; the ursane-type triterpenoid) (Figure 10a). The latter one seems to be studied more intensively. Based on the recent review papers dealing with the boswellic acids, these natural products are biologically and/or pharmacologically active in the treatment of diabetes mellitus [83,84,85,86], rheumatoid arthritis, and other inflammations [83,87], and in the treatment of the viral infections and cancers [88,89,90].

Among the most recent articles, still not covered by any review paper, a study dealing with radiotherapy skin care appeared as an important course of investigation focused on the boswellic acids [91]. The main factor damaging skin during radiotherapy is represented by free radicals forming granulocytes with water molecules in the inflamed area. The application of substances with antioxidant properties, including plant extracts rich in antioxidants, seems to be a novel therapy in radiodermatitis treatment [91]. A series of cosmetic preparations containing additives of plant origin, i.e., those obtained from the *Boswellia* species, were prepared, investigated, and well described in the original literature [91].

Another paper described how chronic inflammatory diseases can also be treated by the oleo-gum resin extract from *Boswellia serrata* [92]. The study evaluated the human pharmacokinetics of a hybrid-hydrogel formulation of *Boswellia* extract standardized for both the volatile and the non-volatile biologically active ingredients in a comparison with the unformulated extract [92]. Because *Boswellia serrata* has been known as an Ayurvedic herb used in traditional medicine, the investigation generally proved the biological and/or the pharmacological importance of the studied hybrid-hydrogel formulation for an efficient treatment of the inflammation.

Drug delivery systems and the formation of nano-assemblies for different types applied for treating important diseases, namely cancers, were investigated with the boswellic acids and their derivatives as well [93]. The investigation aimed to develop a novel delivery system for 3-*O*-acetyl-11-keto-β-boswellic acid (β-AKBA; **10c**; Figure 10a) using the nanoparticles composed of chitosan, sodium alginate, and calcium chloride (CS-SA-CaCl_2_) for targeting colorectal cancer [93]. This investigation introduced the CS-SA-CaCl_2_ nanoparticles as a novel delivery system for β-AKBA (**10c**) with the potential for cytotoxicity. By addressing the limitations of the conventional chemotherapy, the research resulted in a finding of an effective drug delivery system capable of enhancing the therapeutic efficacy of these derivatives in colorectal cancer treatment [93].

Other applications of the nano-formulations of β-AKBA (**10c**) were studied in connection with the antioxidant, antimicrobial, and antifungal effects of the target boswellic acid derivatives [94,95,96]. There is an imperative and rising need for novel antimicrobial agents, namely due to the increasing number of clinical bacterial strains resistant to one or more antibiotics. The promising antibacterial agents have often been obtained from natural sources. However, their limitations in terms of a low solubility in physiological medium, a low bioavailability, and a low biological action, as well as the stability issues and a readily destructive tendency, have restricted their use as medications in a health context. The spherical and uniformly sized nanoparticles had a sustained diffusion-controlled drug release that could last up to 24 h. Boswellic acid coupled with the chitosan-based nanoparticles displayed more potent antimicrobial and antifungal effects [94,95,96]. The antioxidant characteristics were also enhanced when the biologically active components were applied in the form of nanoparticles [94,95,96].

One of the most recent papers dealt with the less frequently studied α-boswellic acid (**10a**; Figure 10a) [97]. The authors studied the acceleration of the wound healing both in vivo and in vitro using α-boswellic acid as the therapeutic agent [97]. α-Boswellic acid demonstrated a relatively favorable pharmacological performance [97]. The half-life of α-boswellic acid was six hours, and the steady-state levels were achieved approximately 30 h post-treatment [97]. The authors described how several signaling pathways have been implicated in wound healing [97]. The Notch signaling pathway has demonstrated the beneficial effects on the endothelial, keratinocyte, and fibroblast cells during the wound healing process [97]. The activation of the Nrf2 signaling pathway was associated with accelerated wound healing through the attenuation of cellular stress and the augmentation of the cellular antioxidant capacity. A significant influence of α-boswellic acid was suggested on cyclin D1, a critical NF-κB target gene, and the NF-κB signaling pathway is crucially involved in the wound healing process [97]. Therefore, it was identified as a potential mechanism underlying the effect of α-boswellic acid (**10a**) in the given investigation [97]. The activity of the NF-κB pathway was significantly modulated by α-boswellic acid (**10a**). The performed investigation confirmed the impact of **10a** through the NF-κB pathway [97]. However, this study also demonstrated some limitations. While the previous clinical trials have predominantly focused on other types of the boswellic acid derivatives and on other inflammatory diseases, only animal models were applied in this study [97]. Therefore, additional rigorous clinical trials focused on **10a** in acute wound healing would be essential to validate its safety and its efficacy [97]. While the reviewed study specifically investigated fibroblasts, numerous other cell types, including neutrophils, macrophages, and endothelial cells, may also be impacted by **10a**. Finally, this study was limited to the detection of a few common cytokines and proteins, which may not provide a complete evaluation of **10a** [97]. Therefore, a comprehensive proteomic analysis could be advised to provide more detailed evidence for comprehending the pathological and the physiological changes, and the mechanisms underlying the wound healing process [97].

Recently, several new papers have appeared, dealing with the investigation of cytotoxicity of different derivatives of the boswellic acids [97,98,99]. One of the most recent papers focused on the investigation of carboplatin, a potent chemotherapeutic agent, the effectiveness of which was constrained by its side effects. The effects of carboplatin (**Cp**) were compared to those of an oleo-gum resin from the *Boswellia sacra* tree that demonstrated cytotoxicity in the cancer cells. The investigation explored the synergistic potential of the nanoparticles formulated from the *Boswellia sacra* methanolic extract (**BME**), to enhance the therapeutic efficacy of the carboplatin at reduced doses [98]. The **BME** contained three compounds with high intensities, maslinic acid (**5a**; Figure 5), 2,2′-methylene-bis(6-*tert*-butyl-4-methylphenol), and β-AKBA (**10c**; Figure 10a), all of them being known to display cytotoxicity [100,101]. The nanoparticles (**BME NPs**) were prepared, loaded with the carboplatin (**Cp**), and coated with the positively charged chitosan (**CS**) for an enhanced cell interaction, yielding nanoparticles (**Cp@CS/BME NPs**) with an average size of 160.2 ± 4.6 nm and a zeta potential of 12.7 ± 1.5 mV [98]. The in vitro studies in the HT-29 and the Caco-2 colorectal cancer cell lines demonstrated the ability of the nano-formulations to increase the carboplatin (**Cp**) uptake significantly, and thus to enhance cytotoxicity (Table 10a) [98]. The apoptosis assays further confirmed an increased induction of cell death with the nanoparticles (**Cp@CS/BME NPs**). The cell cycle analysis revealed that treatment with the nanoparticles (**Cp@CS/BME NPs**) led to a significant increase in the sub-G1 phase, indicative of enhanced apoptosis, and a marked decrease in the G1-phase population coupled with an increased G2/M-phase arrest in both cancer cell lines (HT-29 and Caco-2) [98]. Further gene expression analysis demonstrated a substantial downregulation of the anti-apoptotic gene Bcl-2, and an upregulation of the pro-apoptotic genes Bax, PUMA, and BID, following treatment with the studied nanoparticles (**Cp@CS/BME NPs**) [98]. This investigation presented a promising and innovative strategy for enhancing the therapeutic efficacy of the chemotherapeutic agents using naturally derived ingredients, while limiting the side effects [98].

**Figure 11:**
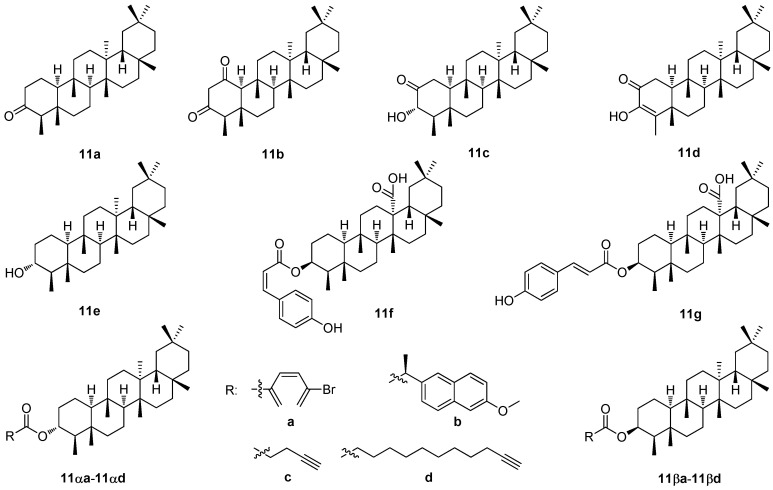
The structures of the different friedelane-type compounds mentioned in this chapter.

A series of 12 new amides (**10baa**–**10bal**; Figure 10b) and 11 new 1*H*-1,2,3-triazole analogs (**10bba**–**10bbk**; Figure 10b) of 3-*O*-acetyl-11-keto-β-boswellic acid (β-AKBA; **10c**; Figure 10a) were prepared. The target products **10bab**–**10bbk** were prepared in high yields (92–96%) by reacting **10baa** with various substituted aromatic azides in the presence of copper(I) iodide. The structures of all new compounds (**10baa**–**10bal** and **10bba**–**10bbk**) were confirmed by analyzing their relevant analytical data, i.e., their ^1^H NMR and ^13^C NMR spectra, and the HRMS, and—where appropriate—^19^F NMR spectra as well [99]. The human breast cancer (MDA-MB-231) growth inhibitory activities of all target compounds were screened and evaluated [99]. The cytotoxic potential of the synthesized compounds was studied in the triple-negative breast cancer cell line (MDA-MB-231) and in the normal non-malignant cell line (MCF-10A). The synthesized compounds displayed significant anti-proliferative activities (Table 10b) [99]. Among them, compounds **10bba** and **10bbi** showed remarkable effects and were several times more potent than the parent compound (β-AKBA; **10c**). Overall, this study paved the way to designing the potentially important medicinal analogs of **10c** as the anti-breast cancer agents.

Different concentrations (*c* = 2.5, 5, 10, and 20 μM) of **10baa**–**10bal** and **10bba**–**10bbk** were tested in the MDA-MB-231 human breast cancer cell line to evaluate their growth effects and compared with their cytotoxicity values in the MCF-10A human normal breast epithelial cells. The MTT assay was used to determine any decrease in the cancer cell viability induced by the cytotoxic agents (Table 10b). However, this study indicated that compounds **10bba** and **10bbi** could certainly be further optimized to improve the potency and the selectivity in the optimized structures in a future. To suggest a possible way to improve the biological activity, the phenyl and the 4-chlorophenyl moieties of these compounds could be substituted by the pyridyl moiety or by the other heterocyclic ones [99].

The structure–activity relationships of the derivatives of the boswellic acids **10a** and **10b** revealed two types of results. The *Boswellia sacra* methanolic extract and the nanoparticles formed therefrom showed only week cytotoxicity in the HT-29 and the Caco-2 cancer cell lines. However, when this extract was combined with the carboplatin and the nanoparticles were formed from this mixture, cytotoxicity of the formed nanostructured material increased substantially. This example showed clearly the potential advantage of the nanomaterials for augmenting the biological activity values of the biologically and/or pharmacologically important agents in the nanomaterials. In turn, a derivative of **10b**, β-AKBA (**10c**), was the parent compound of a series of the amide derivatives (**10baa**–**10bal** and **10bba**–**10bbk**) tested in the MDA-MB-231 breast cancer cells. Two compounds of this series (**10bba** and **10bbi**) displayed the highest cytotoxicity values of the tested series. However, a majority of the compounds of this series showed better results than the parent compound **10c** did.

## 6. Friedelane Triterpenoids

Friedelin (friedelan-3-one; **11a**; Figure 11) is another type of pentacyclic triterpenoid isolated from various plant species and different plant families, as well as from mosses and lichen [102]. Generally, friedelane triterpenoids were found in the cork tissues and the leaf materials of diverse plant genera, such as Celastraceae, Asteraceae, Fabaceae, and Myrtaceae. They possess many biological effects, including anti-inflammatory, antioxidant, cytotoxic, and antimicrobial activities [102]. Friedelin (**11a**) also shows an anti-insect effect and the ability to alter the soil microbial ecology, making it vital to agriculture [102]. Friedelin (**11a**), displaying low cytotoxicity to normal cells, could be one of the phytochemicals with a priority importance to be employed in novel drug design [102].

The extract of the *Loeseneriella africana* stem and its constituents was recently investigated for its antibacterial, resistance modulation, biofilm inhibition and efflux pump inhibition potential [103]. Its antimicrobial activity was investigated by high-throughput spot culture growth inhibition and broth microdilution assays. A resistance modulation activity was studied using anti-biofilm formation and efflux pump inhibition assays. A purification of the extract was made by chromatography, and the isolated compounds were characterized by the usual analytical methods. The whole extract of *L. africana* and the isolated constituents therefrom displayed antibacterial activity against the tested microorganisms with MIC values found in a range from 62.5 to 500.0 μg·mL^−1^. The whole extract demonstrated a resistance modulation effect through strong biofilm inhibition and efflux pump inhibition activities, namely against *Staphylococcus aureus* ATCC 25923, *Escherichia coli* ATCC 25922, and *Pseudomonas aeruginosa* ATCC 27853. A chromatographic fractionation of the ethyl acetate extract resulted in the isolation of the triterpenoid **11b** (Figure 11) and β-sitosterol [103]. Both of these compounds showed antibacterial activity against *E. coli* and *P. aeruginosa*. They also demonstrated an anti-biofilm formation effect at a concentration range of *c* = 3–100 μg·mL^−1^, and bacterial efflux pump inhibition activity at 1/2 MIC and 1/4 MIC against *E. coli* and *P. aeruginosa*, respectively. The results justified the indigenous applications of *L. africana* for managing microbial infections, and showed the antimicrobial potency of the friedelane triterpenoid **11b** (Figure 11) [103].

Column chromatography of the stem bark extracts of *Pterocarpus anglonesis* resulted in the isolation and structure elucidation of seven compounds, which included friedelan-3-one (**11a**), 3α-hydroxyfriedel-2-one (**11c**), 3-hydroxyfriedel-3-en-2-one (**11d**), lup-20(29)-en-3-ol, stigmasta-5-22-dien-3-ol, (3β)-3-acetoxyolean-12-en-28-oic acid, and tetradecyl (*E*)-ferulate (Figure 11) [104]. *P. anglonesis* is an indigenous medicinal plant belonging to the *Pterocarpus* genus of the Fabaceae family. It has been used to treat stomach problems, headaches, malaria, mouth ulcers, blackwater fever, gonorrhea, ringworm, diarrhea, and heavy menstruation, and to stimulate breast milk production. The triple-negative breast cancer (HCC70), hormone receptor-positive breast cancer (MCF-7), and non-malignant mammary epithelial cell lines (MCF-12A) were used to test cytotoxicity of the isolated compounds. Overall, the compounds showed either no or very low cytotoxicity in all three cell lines tested [104].

While **11a** and **11c** (Figure 11) were found to display no cytotoxicity in the cancer cell lines HCC70 and MCF7, they showed cytotoxicity in the non-malignant cell line MCF12A. In turn, cytotoxicity was also found with **11d** (Figure 11) in the MCF7 cancer cell line (IC_50_ = 100.8 μM) [104].

Nine pentacyclic triterpene derivatives were isolated from the leaves of *Camellia hakodae* Ninh. [105]. The extracts were made by several different solvents, from which only the extracts made either by dichloromethane (CH-C) or by ethyl acetate (CH-E) showed low cytotoxicity (Table S2; Supplementary Material). The extracts were analyzed, and their constituents were identified. A new 3-*O*-*cis*-*p*-coumaroyl trichadenic acid B and two new ursane-type triterpene derivatives, 11α,12-[1-(methyl)-2-(4-hydroxy-3-methoxyphenyl)ethane-1,2-dioxy]-urs-12-ene-3β-ol and 11α,12-[2-(methyl)-1-(4-hydroxy-3-methoxyphenyl)ethane-1,2-dioxy]-urs-12-ene-3β-ol, along with the six known compounds were found. Three of them (**11e**, **11f,** and **11g**; Figure 11) belong among the friedelane triterpenoids [105]. This was the first report on the pentacyclic triterpenoids from this plant species [105]. Cytotoxicity of the new compounds was tested in the four human cancer cell lines (KB; Hep-G2; Lu; and MCF-7) using the MTT assay. The new isolated compounds showed cytotoxicity values much lower than those displayed by the positive reference (Table S2; Supplementary Material) [105].

Friedelanes were reported to have a broad spectrum of biological activities, namely, cytotoxic, anti-inflammatory, or anti-nociceptive effects [106]. As usual in the investigation of triterpenoids, their structural modifications can enhance their biological activity and their selectivity, while improving their physicochemical and pharmacokinetic aspects [106]. Recently, eight novel esters modifying the friedelane skeleton were synthesized [106]. Four of them were the derivatives of 3α-friedelinol, i.e., 3α-friedelan-3-yl *p*-bromobenzoate (**11αa**), 3α-friedelan-3-yl naproxenate (**11αb**), 3α-friedelan-3-yl 4-pentynoate (**11αc**), and 3α-friedelan-3-yl 10-undecynoate (**11αd**). Four other compounds were derivatives of 3β-friedelinol, i.e., 3β-friedelan-3-yl *p*-bromobenzoate (**11βa**), 3β-friedelan-3-yl naproxenate (**11βb**), 3β-friedelan-3-yl 4-pentynoate (**11βc**), and 3β-friedelan-3-yl 10-undecynoate (**11βd**) (Figure 11). Overall, 3α-friedelinol (**11αa**) showed greater reactivity when compared with the β-epimer (**11βb**). The esters **11αb**, **11αc**, **11αd**, **11βb**, and **11βc** were tested for their potential anti-leukemic activity in the THP-1 and K-562 cells (Table S3; Supplementary Material) [106]. All tested compounds showed very low cytotoxicity in both cell lines. Compound **11βb** (IC_50_ = 266 ± 6 μM) was the most active compound in the THP-1 cells, and **11αc** (IC_50_ = 267 ± 5 μM) was the most active against the K-562 cells (Table S3; Supplementary Material) [106].

The structure–activity relationships of the compounds derived from the friedelane triterpenoids revealed that their biological effects were generally too low for these compounds to have any practical applicability. However, a challenge always exists to design more potent derivatives of the friedelane triterpenoids in the future to discover a field of biological activity in which these triterpenoids and their derivatives could show their potential.

## 7. Evaluation, Conclusions, and Future Challenges

It is almost impossible to make an evaluative summary of the structure–activity relationships of the pentacyclic triterpenoids and their derivatives mentioned in this review paper. The reason consists in the fact that the different authors focused on different biological effects of the studied compounds. Even if they focused on the investigation of cytotoxicity, they used various cancer cell lines, and due to this fact, the achieved results could hardly be compared with each other. Therefore, just a short summary presented here, based on the partial structure–activity relationship analyses mentioned at the end of each chapter.

Thus, compound **2f** was the most active of the series of the phenyl acetylene and phenyl isoxasole derivatives of arjunolic acid (**1g**) in the study of the α-glucosidase and tyrosinase inhibitory activity. Compounds **3i** and **3r** (the arjunolic acid acetals), and compound **4qb** (the arjunolic acid derivative containing a pentameric A-ring with an enal moiety) were evaluated as other derivatives of arjunolic acid (**1g**) being of priority importance to be subjected to a detailed investigation of the cytotoxicity effects.

Among the sulfamated derivatives of maslinic and asiatic acid, the only compound, **5d,** derived from asiatic acid (**1e**) inhibited the carbonic anhydrase isoform *h*CA VA with activity comparable to the positive reference compound (acetazolamide). A series of ionic derivatives of maslinic acid (**5a**) and corosolic acid (**6a**) resulted in a finding that several compounds of this series (**6b**–**6g**) showed considerable cytotoxicity effects in the MCF7, Jurkat, and THP-1 cancer cell lines. The investigation of the cytotoxicity of the quinoline and isoquinoline amide derivatives (**7a**–**7k**) of corosolic acid (**6a**) revealed that **7b** was the most active compound in treating the HT-29 cancer cell line, showing a cytotoxicity value comparable to that of doxorubicin.

The structure–activity relationship analysis of the derivatives of moronic acid (**8a**) and morolic acid (**8b**) revealed that compounds **8k** (the derivative of morolic acid bearing the piperazine motif combined with that of l-methionine) and **9h** (the derivative of morolic acid combined with the tripeptide MAG) showed acceptable cytotoxicity in several cancer cell lines (CCRF-CEM, HeLa, MCF7, and G-361). In addition, compound **9d** (the derivative of moronic acid combined with the tripeptide MAG) inhibited *S. aureus* at 100%, and compounds **9g**, **9i,** and **9j** (the derivatives of morolic acid combined with the tripeptides GAM or MAG) showed anti-HIV-1 and/or anti-HSV-1 activity with selectivity index values comparable to those of **8a** and **8b** or even better than the selectivity index values of **8a** and **8b**.

The *Boswellia sacra* methanolic extract and the nanoparticles formed therefrom showed only weak cytotoxicity in the HT-29 and the Caco-2 cancer cell lines. However, when this extract was combined with carboplatin, and the nanoparticles were formed from this mixture, the cytotoxicity values of the formed nanostructured material increased substantially. This example showed clearly the potential advantage of the nanomaterials for augmenting the biological activity values of the biologically important agents in nanomaterials. Among the derivatives of β-AKBA (**10c**), two compounds (**10bba** and **10bbi**; the new 1*H*-1,2,3-triazole derivatives of **10c**) displayed the highest cytotoxicity values of the tested series, which were better than the results displayed by their parent compound **10c**.

The friedelane triterpenoids revealed that their biological effects were generally too low for these compounds to have any practical applicability so far. The future challenge is to design compounds with an enhanced ability to contribute to the biological application of the friedelane triterpenoids.

Nevertheless, many compounds reviewed here were proven to display high biological activity of different types, namely cytotoxicity, antimicrobial, or antiviral activity, etc. Therefore, further development in the investigation of the different structural modifications of the natural molecules is a challenging and important task in designing novel molecules with potentially enhanced biological characteristics. This reviewed series of pentacyclic triterpenoids has important potential to result in successes such as those achieved with the betulinic acid-based agents, of which several have already been used in pharmacological and/or medicinal practice. Among them, the antiviral agent bevirimat [107,108,109] and its more recently designed and structurally analogous molecules [110,111,112] should be pointed out. Another achievement resulted from investigating a mixture of several birch triterpenoids representing the active constituents of the recently introduced botanical drug for treating epidermolysis bullosa (oleoegel-S10) [113].

## Figures and Tables

**Table 1:** The nitric oxide (NO) inhibition effects in the LPS-activated RAW264.7 cells caused by **1a**–**1i** [42].

Compound	NO Inhibition, IC_50_ ± SD [μM] (n = 3, *p* < 0.05)	Compound	NO Inhibition, IC_50_ ± SD [μM] (n = 3, *p* < 0.05)
**1a**	37.57 ± 1.88	**1f**	22.69 ± 1.58
**1b**	26.04 ± 1.45	**1g**	18.79 ± 0.95
**1c**	20.55 ± 0.35	**1h**	19.21 ± 0.83
**1d**	25.37 ± 1.36	**1i**	22.01 ± 0.72
**1e**	8.91 ± 0.36	Dexamethasone ^a^	14.05 ± 1.17

^a^ The positive reference compound.

**Table 2:** The α-glucosidase and the tyrosinase inhibitory activity of arjunolic acid (**1g**), its new derivatives (**2a**–**2f**), and the reference compounds [43].

Compound	α-Glucosidase Inhibitory Activity (IC_50_ ± SD [µM])	Tyrosinase Inhibitory Activity (IC_50_ ± SD [µM])
**1g**	>600	85.2 ± 4.01
**2a**	588.8 ± 3.84	58.1 ± 5.9
**2b**	300.0 ± 0.95	30.6 ± 4.6
**2c**	425.0 ± 0.97	35.4 ± 15.3
**2d**	63.7 ± 0.52	25.2 ± 7.7
**2e**	172.0 ± 0.36	14.3 ± 7.6
**2f**	14.5 ± 0.15	15.2 ± 5.6
Reference compound	10.4 ± 0.06 ^a^	41.5 ± 1.0 ^b^

^a^ Reference compound = acarbose. ^b^ Reference compound = kojic acid.

**Table 3:** The IC_50_ values of the compounds **3b**, **3d**, **3i,** and **3r** in the selected cell lines of the different cancers [44].

Cancer	Subpanel	Cytotoxicity of the Compounds 3b, 3d, 3i, and 3r (IC_50_ [µM])			
		3b	3d	3i	3r
Colon cancer	COLO205	4.47	8.51	3.47	0.18
Melanoma	MALME-3M	14.50	19.50	11.20	0.54
	M14	12.30	18.20	6.92	0.90
	MDA-MB-435	10.00	17.40	6.31	0.80
	SK-MEL-5	5.37	7.41	5.89	0.46
	UACC-62	12.90	20.90	13.20	0.70
Renal cancer	ACHN	4.37	5.75	3.98	0.47
Breast cancer	MDA-MB-468	10.50	13.80	7.59	0.69

**Table 4:** (**a**)**.** Cytotoxicity of arjunolic acid (**1g**) and its derivatives (**4a**–**4sb**) in the PANC-1 and the HT-29 cancer cell lines [45] ^a^. (**b**). Cytotoxicity of compounds **4pb**–**4sb** in several cancer cell lines and in the non-malignant human fibroblasts (BJ) [45] ^A^.

**(a)**					
**Compound**	**Cell Line, IC_50_ ± SD [µM] ^b^**		**Compound**	**Cell Line, IC_50_ ± SD [µM] ^b^**	
	**PANC-1 ^c^**	**HT-29 ^d^**		**PANC-1 ^c^**	**HT-29 ^d^**
**1g**	56.31 ± 8.61	36.42 ± 1.88	**4na**	16.65 ± 0.17	25.92 ± 1.46
**4a**	14.90 ± 0.71	12.43 ± 1.78	**4oa**	22.03 ± 1.69	N. D.
**4b**	3.63 ± 0.44	19.26 ± 0.50	**4pa**	6.70 ± 0.38	5.59 ± 0.33
**4c**	0.93 ± 0.23	16.53 ± 4.50	**4qa**	7.14 ± 0.60	6.70 ± 0.65
**4d**	0.63 ± 0.06	1.17 ± 0.18	**4ra**	4.58 ± 0.32	4.46 ± 0.37
**4e**	>30	>30	**4sa**	4.92 ± 0.32	4.38 ± 0.34
**4f**	>30	>30	**4lb**	5.27 ± 0.27	5.73 ± 0.98
**4g**	27.57 ± 2.62	25.48 ± 2.32	**4mb**	3.74 ± 0.25	5.81 ± 0.19
**4h**	19.65 ± 1.33	16.73 ± 1.06	**4nb**	2.38 ± 0.42	7.41 ± 0.70
**4i**	20.20 ± 1.19	11.60 ± 0.26	**4ob**	4.15 ± 0.38	3.43 ± 0.12
**4j**	20.25 ± 1.56	13.70 ± 0.42	**4pb**	0.82 ± 0.04	0.87 ± 0.03
**4k**	18.95 ± 1.35	12.83 ± 0.87	**4qb**	0.47 ± 0.04	0.51 ± 0.02
**4la**	>30	>30	**4rb**	0.43 ± 0.02	0.55 ± 0.04
**4ma**	29.32 ± 1.14	N. D.	**4sb**	0.84 ± 0.05	0.55 ± 0.04
**(b)**					
**Compound**	**Cell Line, IC_50_ ± SD [μM] ^B^**				
	**Melanoma**		**Lung**		**Fibroblasts**
	**A375 ^C^**	**SK-MEL-28 ^D^**	**A-549 ^E^**	**H-2170 ^F^**	**BJ ^G^**
**4pb**	0.85 ± 0.04	0.85 ± 0.09	1.22 ± 0.03	2.11 ± 0.09	---
**4qb**	0.58 ± 0.04	0.58 ± 0.02	0.83 ± 0.01	1.33 ± 0.03	3.06 ± 0.23
**4rb**	0.55 ± 0.02	0.45 ± 0.02	0.58 ± 0.04	1.30 ± 0.03	2.79 ± 0.08
**4sb**	0.59 ± 0.06	0.53 ± 0.02	0.82 ± 0.02	1.51 ± 0.14	---

^a^ The PANC-1 and the HT-29 cells were treated with increasing concentrations of each compound for 72 h. The IC_50_ values were determined by the MTT assay and were represented as the mean (±SD) of three independent experiments. N. D. = not determined. ^b^ The IC_50_ is the concentration of the compound inhibiting 50% of the cell growth. ^c^ PANC-1 is a human pancreatic cancer cell line. ^d^ HT-29 is a human colon cancer cell line (colorectal adenocarcinoma). ^A^ The different cell lines were treated with increasing concentrations of each compound for 72 h. The IC_50_ values were determined by the MTT assay in all cancer cell lines. The IC_50_ values are expressed as a mean (±SD) of three independent experiments. ^B^ The IC_50_ is the concentration of the compound that inhibits 50% of cell growth. ^C^ Human melanoma cell line. ^D^ Human melanoma cell line. ^E^ Human adenocarcinoma. ^F^ Lung carcinoma. ^G^ Human fibroblasts.

**Table 5:** Inhibition of the carbonic anhydrase isoforms *h*CA I, *h*CA II, *h*CA VA, and *h*CA IX [56].

Compound	Inhibition of the Human Carbonic Anhydrase Isoforms (Ki [nM])			
	*h*CA I ^a^	*h*CA II ^a^	*h*CA VA ^a^	*h*CA IX ^a^
**5b**	925.0	390.4	457.0	95.2
**5c**	3781.0	507.7	362.3	1923.0
**5d**	5230.0	297.5	36.2	1126.0
AAZ ^b^	250.0	12.1	63.0	25.8

^a^ The values (K_i_ [nM]) were obtained as the means from three different assays by a stopped flow technique; errors were in the range of ±5–10% of the reported values. ^b^ AAZ (acetazolamide) was used as a positive reference compound.

**Table 6:** The in vitro cytotoxicity of compounds **5a** and **6a**–**6g** [60].

Compound	Cytotoxicity (IC_50_ ± SD [μM]) After 48 h of Incubation with the Cells ^a^						
	MCF-7	HTC116	A549	HepG2	Jurkat	THP-1	HEK293
**5a**	60.13 ± 0.82	48.22 ± 5.62	65.58 ± 4.90	74.37 ± 3.61	23.44 ± 1.45	43.14 ± 2.06	45.38 ± 1.87
**6a**	35.85 ± 2.39	28.86 ± 2.22	46.53 ± 1.13	51.40 ± 2.21	15.33 ± 0.79	19.27 ± 0.07	21.50 ± 0.41
**6b**	0.53 ± 0.12	2.97 ± 0.85	7.32 ± 1.74	19.12 ± 3.44	1.71 ± 0.30	1.18 ± 0.14	3.23 ± 0.61
**6c**	0.52 ± 0.27	2.15 ± 0.71	5.37 ± 0.86	6.90 ± 0.34	0.72 ± 0.10	0.75 ± 0.07	1.97 ± 0.76
**6d**	1.35 ± 0.49	4.48 ± 1.12	10.10 ± 1.79	30.61 ± 1.79	3.66 ± 0.51	2.29 ± 0.25	8.26 ± 0.38
**6e**	0.55 ± 0.19	1.55 ± 0.65	4.91 ± 1.12	7.74 ± 0.56	0.64 ± 0.07	0.94 ± 0.03	1.32 ± 0.77
**6f**	1.99 ± 0.29	2.70 ± 0.86	8.93 ± 1.43	19.10 ± 0.34	3.92 ± 0.27	2.16 ± 0.20	4.74 ± 0.83
**6g**	1.62 ± 0.67	1.46 ± 0.79	4.28 ± 1.03	4.22 ± 0.37	0.73 ± 0.22	0.81 ± 0.03	2.01 ± 0.64

^a^ The data are presented as the mean IC_50_ ± SD, and the number of the experimental repetitions is three. The differences between IC_50_ values in the cancer cells (MCF-7, HTC116, A549, HepG2, Jurkat, and THP-1) and the IC_50_ value in the non-malignant HEK293 cells are statistically significant.

**Table 7:** The SRB cytotoxicity assay performed with the compounds **7a**–**7k** [63].

Compd.	The SRB Cytotoxicity Assay, the IC_50_ ± SD Values [μM] After 72 h of Treatment ^a^									
	A2780 ^b^	A2780cis ^b^	A549 ^b^	HT29 ^b^	MCF7 ^b^	CCD18Co ^b^	RI ^c^	SI1 ^c^	SI2 ^c^	SI3 ^c^
**7a**	2.45 ± 0.47	7.32 ± 1.37	3.95 ± 0.59	1.44 ± 0.15	3.95 ± 2.71	25.6 ± 13.7	2.99	10.4	3.5	17.8
**7b**	1.25 ± 0.23	2.51 ± 0.31	1.26 ± 0.25	0.22 ± 0.09	1.95 ± 0.65	6.02 ± 2.65	2.01	4.8	2.4	7.4
**7c**	2.80 ± 0.81	6.07 ± 2.12	7.46 ± 1.23	1.34 ± 0.28	4.66 ± 2.70	>100.0	2.17	35.7	16.5	74.6
**7d**	4.32 ± 0.34	6.39 ± 0.85	7.66 ± 2.06	2.13 ± 0.40	7.01 ± 0.96	46.6 ± 21.2	1.48	10.8	7.3	21.9
**7e**	24.3 ± 3.64	50.6 ± 11.6	30.9 ± 8.87	12.5 ± 7.12	30.1 ± 2.58	>100.0	2.08	4.1	1.9	8.0
**7f**	6.04 ± 1.25	18.2 ± 7.76	6.20 ± 1.79	4.80 ± 0.78	11.8 ± 2.60	55.4 ± 33.3	3.01	9.2	3.1	11.5
**7g**	2.86 ± 0.43	6.06 ± 1.15	4.72 ± 0.76	1.21 ± 0.33	3.48 ± 2.00	8.16 ± 4.18	2.12	2.8	1.3	6.7
**7h**	3.95 ± 0.36	8.43 ± 2.68	5.01 ± 0.44	2.53 ± 0.72	4.41 ± 2.14	40.8 ± 8.25	2.13	10.3	4.8	16.1
**7i**	4.71 ± 1.27	10.9 ± 4.83	9.91 ± 2.13	3.96 ± 1.28	6.40 ± 0.89	24.8 ± 19.3	2.31	5.3	2.3	6.3
**7j**	4.34 ± 0.66	11.4 ± 5.24	10.1 ± 2.13	2.31 ± 0.85	6.64 ± 1.60	>100.0	2.63	23.1	8.8	4.3
**7k**	3.74 ± 0.19	9.46 ± 4.36	3.59 ± 0.78	1.71 ± 0.54	3.56 ± 0.31	>100.0	2.53	26.7	10.6	58.5
Doxorubicin	0.013	0.127	0.08	0.11	0.029	0.321	9.77	24.7	2.5	2.9

^a^ The values are the average from three to four independent experiments, except for doxorubicin, used as a positive reference, where n = 1. ^b^ Human cancer cell lines: A2780 (ovarian carcinoma), A2780cis (resistant derivative of A2780), A549 (lung carcinoma), HT29 (colorectal carcinoma), MCF7 (breast carcinoma), and CCD18Co (non-malignant human fibroblasts). ^c^ Resistance index (RI): IC_50_ ratio of A2780cis/A2780, selectivity index 1 (SI1): IC_50_ ratio of CCD18Co/A2780, selectivity index 2 (SI2): IC_50_ ratio of CCD18Co/A2780cis; and selectivity index 3 (SI3): IC_50_ ratio CCD18Co/HT29.

**Table 8:** The cytotoxicity screening tests (IC_50_ ± SD [µM], 72 h) of the prepared compounds (**8c**–**8n**), and their parent triterpenoids **8a** and **8b** in three cancer cell lines and in the normal human fibroblasts [79].

Compound	IC_50_ ± SD [µM] After 72 h ^a^			
	CCRF-CEM ^b^	HeLa ^c^	G-361 ^d^	BJ ^e^
**8a**	38.3 ± 0.4	35.1 ± 1.7	42.7 ± 3.3	>50
**8b**	38.6 ± 0.3	47.7 ± 1.8	>50	>50
**8d**	36.5 ± 4.5	8.5 ± 0.3	7.4 ± 0.3	10.8 ± 0.8
**8e**	21.4 ± 0.7	7.7 ± 0.3	7.0 ± 0.8	9.2 ± 1.2
**8f**	12.2 ± 0.8	7.8 ± 0.2	7.2 ± 0.4	8.7 ± 0.5
**8g**	>50	27.3 ± 4.7	>50	>50
**8j**	19.3 ± 1.5	8.3 ± 0.5	6.6 ± 0.1	10.9 ± 3.1
**8k**	11.7 ± 2.4 ^f^	9.0 ± 0.7 ^g^	10.6 ± 5.5 ^h^	43.3 ± 1.5
**8n**	44.3 ± 0.3	33.3 ± 4.4	>50	>50
**CDDP** ^i^	0.8 ± 0.1	11.4 ± 3.8	4.5 ± 0.6	6.9 ± 0.9

^a^ Compounds **8c**, **8h**, **8i**, **8l,** and **8m** displayed an IC_50_ > 50 µM in all four cell lines; ^b^ T-lymphoblastic leukemia; ^c^ cervical carcinoma; ^d^ malignant melanoma; ^e^ normal human fibroblasts; ^f^ selectivity index, SI = 3.9; ^g^ selectivity index, SI = 4.8; ^h^ selectivity index, SI = 4.4; and ^i^ *cis*-diamminedichloroplatinum(II), cisplatin, a pharmacologically used agent for treating cancers, used as a positive reference compound.

**Table 9:** The antimicrobial, antiviral, and cytotoxicity effects of **8a**, **8b,** and their selected derivatives [80].

Compd.	Inhibition of *S. aureus* and *Ent. faecalis* in the dilution test by 8a, 8b, and 9d [%].					
	Inhibition of *S. aureus* in the Dilution Test [%].			Inhibition of *Ent. faecalis* in the Dilution Test [%].		
	250 μM	125 μM	62.5 μM	250 μM	125 μM	62.5 μM
**8a**	95.11	98.43	98.73	96.90	99.06	98.22
**8b**	10.49	25.20	21.43	65.71	71.72	72.31
**9d**	100.00	99.95	99.57	85.02	76.39	22.39
	**The anti-HIV-1 activity and cytotoxicity of the compounds 8a, 8b, 9g, 9i, and 9j in the MT-4 cells, and their anti-HSV-1 activity and cytotoxicity in the Vero cells.**					
	**The anti-HIV-1 activity (EC_50_ ± SD [μM]) and cytotoxicity (CC_50_ ± SD [μM]) in the MT-4 cells.**			**The anti-HSV-1 activity (EC_50_ ± SD [μM]) and cytotoxicity (CC_50_ ± SD [μM]) in the Vero cells.**		
	**EC_50_**	**CC_50_**	**SI**	**EC_50_**	**CC_50_**	**SI**
**8a**	6.3 ± 0.28	53.0 ± 1.7	>8.4	11.0 ± 1.1	~26	~2.4
**8b**	6.4 ± 0.82	>100	>16	12.0 ± 0.51	35.0 ± 5.5	>2.9
**9g**	17.8 ± 2.1	41.0 ± 5.2	>2.3	34.0 ± 3.7	>100	>2.9
**9i**	42.0 ± 4.6	~60	~1.4	27.7 ± 3.5	>100	>3.6
**9j**	12.6 ± 0.82	38.0 ± 4.2	>2.9	30.9 ± 3.3	>100	>3.2
	**Cytotoxicity (IC_50_ ± SD [μM], 72 h) displayed by 8a, 8b, and 9h.**					
	**CCRF-CEM**	**MCF7**	**HeLa**	**G-361**	**BJ**	**SI**
**8a**	38.3 ± 0.4	>50	35.1 ± 1.7	42.7 ± 3.3	>50	
**8b**	38.6 ± 0.3	49.4 ± 0.9	47.7 ± 1.8	>50	>50	
**9h**	12.0 ± 3.3	8.6 ± 0.2	7.9 ± 2.1	8.0 ± 0.6	>50	SI values ^a^

^a^ SI (CCRF-CEM) > 4.0; SI (MCF7) > 6.0; SI (HeLa) > 6.5; and SI (G-361) > 6.3.

**Table 10:** (**a**). Cytotoxicity of **BME**, **BME NPs**, **Cp**, and **Cp@CS/BME NPs** in the CCD 841 CoN normal colon epithelial cell line and the selectivity index (SI) for the HT-29 and the Caco-2 cancer cell lines [98]. (**b**). Cytotoxicity (IC_50_ ± SD [μM]) in the MDA-MB-231 breast cancer cells [99] ^A^.

**(a)**					
**Sample**	**CC_50_ ^a^**	**SI for HT-29 ^b^**		**SI for Caco-2 ^c^**	
**BME**	296.03 ± 3.99	3.17		3.42	
**BME NPs**	190.00 ± 6.98	5.79		7.45	
**Cp**	32.21 ± 1.97	2.02		2.36	
**Cp@CS/BME NPs**	40.69 ± 2.29	13.00		27.31	
**(b)**					
**Comp.**	**IC_50_ [μM]**	**Comp.**	**IC_50_ [μM]**	**Comp.**	**IC_50_ [μM]**
**10baa**	12.2 ± 0.4	**10bai**	16.1 ± 0.8	**10bbe**	12.1 ± 0.4
**10bab**	16.4 ± 0.8	**10baj**	13.2 ± 0.5	**10bbf**	11.2 ± 0.4
**10bac**	18.5 ± 0.4	**10bak**	15.1 ± 0.3	**10bbg**	10.6 ± 0.2
**10bad**	17.2 ± 0.6	**10bal**	17.4 ± 0.6	**10bbh**	12.4 ± 0.8
**10bae**	10.5 ± 0.2	**10bba**	6.4 ± 0.2	**10bbi**	8.1 ± 0.2
**10baf**	9.5 ± 0.6	**10bbb**	10.1 ± 0.4	**10bbj**	14.4 ± 0.2
**10bag**	13.2 ± 0.5	**10bbc**	10.5 ± 0.2	**10bbk**	11.2 ± 0.4
**10bah**	15.1 ± 0.4	**10bbd**	13.5 ± 0.2	**10c** (β-AKBA)	17.27 ± 1.6

^a^ Cytotoxicity in the CCD 841 CoN normal cell line (μg·mL^−1^), presented as an average ± SD (n = 3). ^b^ The selectivity index calculated as the CC_50_/IC_50_ ratio in the HT-29 cells. ^c^ The selectivity index calculated as the CC_50_/IC_50_ ratio in the Caco-2 cells. ^A^ The normal (non-malignant) breast cell line (MCF-10A) was used as the reference (IC_50_ > 20 μM for all compounds tested).

## Data Availability

No new data were created or analyzed in this study. Data sharing is not applicable to this article.

## Supplementary Materials

The following supporting information can be downloaded at https://www.mdpi.com/article/10.3390/molecules30153106/s1: Table S1: The IC_50_ values of the compounds **3b**, **3d**, **3i,** and **3r** in the 60 cell lines of the nine different cancers. Table S2: The in vitro cytotoxicity of the extracts from the leaves of *C. hakodae* extracted with dichloromethane (CH-C) and ethyl acetate (CH-E), and the in vitro cytotoxicity of **11e**, **11f,** and **11g** in four human cell lines [lung (LU), breast (MCF7), epidermoid (KB), and hepatoma (HepG2)]. Table S3: The cytotoxicity values of the compounds **11αb**, **11βb**, **11αc**, **11βc,** and **11αd** obtained from the cell viability assay (MTT) in THP-1 and K-562 cell lines. The cytotoxicity values were compared with those of imatinib or cytarabine.
