# Supplementary material for: Selected Pentacyclic Triterpenoids and Their Derivatives as Biologically Active Compounds
Source: Molecules. 2025 Jul 24;30(15):3106. doi: 10.3390/molecules30153106 (PMC12348783; doi:10.3390/molecules30153106)
Supplement: Supplementary file 1 [file molecules-30-03106-s001.zip › molecules-3671072-supplementary.pdf]

SUPPLEMENTARY MATERIAL

Selected Pentacyclic Triterpenoids and Their Derivatives as Biologically Active Compounds

Zdeněk Wimmer

Department of Chemistry of Natural Compounds, University of Chemistry and Technology in Prague, Technická 5, 16028 Prague, Czech Republic; zdenek.wimmer@vscht.cz or wimmer@biomed.cas.cz

Isotope Laboratory, Institute of Experimental Botany of the Czech Academy of Sciences, Vídeňská 1083, 14220 Prague, Czech Republic

Table S1. The IC_{50} values of the compounds **3b**, **3d**, **3i** and **3r** in the 60 cell lines of the nine different cancers [44].

Cancer	Subpanel	Cytotoxicity of the compounds **3b**, **3d**, **3i** and **3r** (IC_{50} [μM])			
		**3b**	**3d**	**3i**	**3r**
Leukemia	CCRF-CEM	4.47	6.03	5.25	9.33
	HL-60 (TB)	4.37	3.90	4.37	19.90
	K-562	3.39	2.80	3.24	6.30
	MOLT-4	3.31	4.10	4.07	8.30
	RPMI-8226	4.10	9.12	4.79	13.40
	SR	5.30	6.76	6.46	19.05
NSCLC	EKVX	10.50	10.70	7.59	16.90
	HOP-62	7.24	13.80	6.92	15.80
	HOP-92	20.90	41.70	85.10	27.50
	NCI-H226	13.50	22.90	20.90	33.10
	NCI-H23	7.41	10.70	6.46	16.20
	NCI-322M	9.33	13.20	8.71	19.05
	NCI-H460	4.57	5.62	3.02	8.51
	NCI-HS22	9.33	13.80	7.41	20.40
Colon cancer	COLO205	4.47	8.51	3.47	0.18
	HCC-2998	5.75	6.90	5.75	12.50
	HCT-116	3.39	4.20	3.24	6.30
	HCT-15	2.75	3.16	2.82	4.20
	HT-29	4.68	6.76	4.17	18.10
	KM 12	7.41	8.51	7.94	13.40

	SW-620	5.75	9.33	4.37	16.50
CNS cancer	CF-268	15.50	34.70	9.77	25.70
	SF-295	4.47	6.03	4.47	10.00
	SF-539	6.61	14.80	6.17	23.90
	SNB-19	7.41	20.00	7.08	13.50
	SNB-75	21.90	---	---	39.80
	U 251	4.07	52.50	4.37	3.20
Melanoma	LOX IMVI	4.27	5.13	4.17	7.20
	MALME-3M	14.50	19.50	11.20	0.54
	M14	12.30	18.20	6.92	0.90
	MDA-MB-435	10.00	17.40	6.31	0.80
	SK-MEL-2	15.50	20.40	12.00	3.60
	SK-MEL-28	13.50	22.90	12.00	3.80
	SK-MEL-5	5.37	7.41	5.89	0.46
	UACC-257	14.80	24.50	9.77	0.47
	UACC-62	12.90	20.90	13.20	0.70
Ovarian cancer	IGROV-1	8.71	13.20	7.24	19.4
	OVCAR-3	6.03	8.71	5.25	16.90
	OVCAR-4	15.50	24.00	10.00	44.60
	OVCAR-5	17.40	26.90	9.33	29.50
	OVCAR-8	6.61	14.80	5.62	19.40

	NCI/ADR-RES	5.25	8.13	5.13	13.40
	SK-OV-3	13.50	28.20	9.12	60.20
Renal cancer	786-0	7.59	14.80	5.62	33.80
	A498	25.70	28.80	45.70	37.10
	ACHN	4.37	5.75	3.98	0.47
	CAKI-1	6.61	16.60	5.25	36.30
	RXF-393	9.55	26.90	14.50	28.10
	SN 12C	6.92	15.50	5.01	20.40
	TK-10	15.80	55.00	17.80	38.90
	UO-31	7.24	12.00	6.61	16.90
Prostate cancer	PC-3	5.75	7.24	6.76	19.05
	DU-145	11.00	9.55	5.89	21.80
Breast cancer	MCF-7	4.68	5.75	4.27	13.40
	MDA-MB-231/ATCC	19.50	26.30	11.50	30.90
	HS587T	26.30	97.70	---	67.60
	BT-549	17.00	20.90	20.00	23.90
	T-47D	24.50	66.10	22.40	83.10
	MDA-MB-468	10.50	13.80	7.59	0.69

Table S2. The *in vitro* cytotoxicity of the extracts from the leaves of *C. hakodae* made by dichloromethane (CH-C) and ethyl acetate (CH-E), and the *in vitro* cytotoxicity of **11e**, **11f** and **11g** in four human cell lines [lung (LU), breast (MCF7), epidermoid (KB) and hepatoma (HepG2)] [105].

Sample	IC_{50} ± SD [μM]			
	KB	HepG2	LU	MCF7
CH-C	49.6	109.71	73.14	68.2
CH-E	25.6	51.07	53.33	137.84
**11e**	inactive	inactive	inactive	inactive
**11f**	71.25 ± 3.12	71.18 ± 1.35	65.28 ± 3.19	72.96 ± 1.63
**11g**	20.48 ± 1.26	82.73 ± 1.45	23.83 ± 1.94	31.25 ± 2.46
Ellipticine [a]	0.31 ± 0.02	0.56 ± 0.04	0.40 ± 0.05	0.51 ± 0.05

[a] Ellipticine was used as the positive reference.

Table S3. The cytotoxicity values of the compounds **11αb**, **11βb**, **11αc**, **11βc** and **11αd** obtained from the cell viability assay (MTT) in THP-1 and K-562 cell lines. The cytotoxicity values were compared with those of Imatinib or Cytarabine [106].

Compound	IC_{50} ± SD [μM] [a]	
	THP-1 [b]	K-562 [c]
**11αb**	339 ± 8	396 ± 13
**11βb**	266 ± 6	918 ± 43
**11αc**	326 ± 8	267 ± 5
**11βc**	377 ± 36	388 ± 40
**11αd**	572 ± 88	355 ± 30
Imatinib [d]	Not determined	35 ± 4
Cytarabine [d]	41 ± 8	Not determined

[a] The cytotoxicity values (IC_{50} ± SD [μM]) were presented as the means from the two independent experiments; [b] THP-1, acute monocytic leukemia cell line; [c] K-562, chronic myeloid leukemia cell line; [d] the positive references.
